# Nurses’ professional quality of life and job satisfaction in Jordan

**DOI:** 10.3389/fmed.2024.1478316

**Published:** 2024-12-19

**Authors:** Khaldoun M. Hamdan, Rabia S. Allari, Nadiah A. Baghdadi, Shaherah Yousef Andargeery, Abdullah M. Haymour, Evan Sabrah, Abeer M. Shaheen

**Affiliations:** ^1^Faculty of Nursing, Al-Ahliyya Amman University, Amman, Jordan; ^2^Department of Nursing Management and Education, College of Nursing, Princess Nourah Bint Abdulrahman University, Riyadh, Saudi Arabia; ^3^The Specialty Hospital, Amman, Jordan; ^4^Department of Community Health Nursing, School of Nursing, The University of Jordan, Amman, Jordan

**Keywords:** burnout, moral distress, professional quality of life, satisfaction, sustanability, nursing

## Abstract

**Background:**

Professional quality of life has received widespread concern in nursing over the last few years. Nurses with a high professional quality of life enthusiastically approach their work and provide excellent patient care. On the other hand, poor professional quality of life may affect nurses’ quality of care, resulting in job dissatisfaction and jeopardizing patient outcomes.

**Aim:**

To examine the relationship between the professional quality of life and job satisfaction among registered nurses.

**Method:**

A cross-sectional correlational design and convenience sampling were used. Data were collected using the Professional Quality of Life Scale-Health and the Job Satisfaction Survey.

**Results:**

The mean total score for the nurses’ job satisfaction survey was high 117.47 (SD = 27.26). The nature of work subscale had the highest mean, while the fringe benefits subscale had the lowest. The mean total score for the Professional Quality of Life Scale for Health Workers was moderate 98.41 (SD = 12.15). The Compassion Satisfaction subscale had the highest mean score, while the moral distress subscale had the lowest. There was a statistically significant positive relationship between nurses’ job satisfaction and professional quality of life for health workers.

**Conclusion:**

Nursing supervisors need to be more aware of the variables influencing nurses’ professional quality of life and job to assess the degree of moral distress and satisfaction. Although nurses offer their patients physical, psychological, and spiritual care, their duties and interactions with patients can have a negative impact on them. As a result, nurses will be better equipped to care for patients if they have the assistance and support they need.

## Introduction

Among the 65.1 million healthcare workers globally, nurses comprise the largest workforce in the healthcare system, estimated at 27 million (World Health Organization (WHO), 2022). Involving nursing workforce is necessary to increase the quality of healthcare services. Globally, there is a shortage of nurses and midwives, representing more than 50% of the current shortage of health workers. By 2030, the globe will require nine million nurses and midwives for all countries to meet health and well-being (1).

the need to enhance nurses’ quality of working life and satisfaction is growing (2). Besides this challenge, nurses frequently face patient-related complex issues, high and demanding workloads, an aging nursing workforce, ineffective policies supporting nurses in administrative systems, inadequate supervisor support, unfair compensation, unfavorable working conditions, a lack of tools and resources for efficient work, a lack of career and educational opportunities, and unstable work environments (3). These challenging conditions may affect nurses’ quality of life and satisfaction with providing care daily (4).

Professional Quality of Life (PQL) has received widespread concern in nursing over the last few years, especially among nurses in the emergency department, intensive care units, and oncology wards. Helping and caring for patients is relevant to the nursing profession as the core value of nurses, and nurses are the frontline healthcare providers who deliver the most holistic and patient-centered care for patients. PQL refers to an individual’s overall experience of well-being and satisfaction with their job (5). According to the professional quality of life model (5), it is a multidimensional concept encompassing two aspects: compassion satisfaction, which is considered the positive aspect, and compassion fatigue, the negative aspect of professional quality of life. Stamm (5) states that compassion satisfaction involves an individual’s feeling of helping others. Compassion fatigue measures two parts: burnout and secondary traumatic stress. According to Kakemam et al. (6), secondary traumatic stress is less prevalent than burnout, yet it has an intense effect after the occurrence of adverse events on patients. Nurses are prone to burnout because of increased feelings of exhaustion and frustration from not effectively handling their work. On the other hand, nurses are prone to secondary traumatic stress from exposure to patients who suffer from traumatic conditions (5).

Nurses face complex challenges and stressors when they provide patient care, particularly for patients who are at high risk of life-threatening health problems and need cautious or intensive care. The continuous exposure to complex and demanding work environments increases the risk of developing compassion fatigue among nurses (7). These stressful events make nurses more prone to mistakes (8). Scott et al. (9) also mention that daily psychological and physical burdens nurses face can affect their performance and predispose them to error and unanticipated adverse events. In a recent study conducted by Yildirim and Ertem (10), it was found that the monthly number of shifts, manner of work, and average weekly working hours were the factors that affected nurses’ quality of life. Another study conducted by (11) found that nurses’ professional quality of life was affected by COVID-19. Sundgren et al. (12) found that reflective practice was more effective and positively impacted the professional quality of life scores, especially compassion burnout and satisfaction through the mediation of personal and job resources. Self-compassion has been linked with professional quality of life. In addition, it is vital to provide compassionate care to maintain balance (13) and develop resilience against stress and burnout among healthcare providers (14).

Understanding the employees’ work and organizational environment and satisfaction with their presence and involvement in their workplace, resources, and activities influence their professional quality of life (15, 16). A reduction in professional quality of life can decrease the quality of care, patient outcomes, and employees’ motivation while increasing absenteeism, psychological distress, and turnover (17–21). On the other hand, a higher professional quality of life positively influences employees’ performance, organizational identity, job satisfaction, and work commitment (15).

Attention to the factors influencing quality of life will help nurse administrators identify effective strategies to improve organizational efficacy, job satisfaction, and willingness to stay in their jobs. It is crucial to investigate nurses’ perceptions of professional quality of life and its association with job satisfaction, to understand nurses’ experience of compassion satisfaction and compassion fatigue at work. For health organizations to survive, it is essential to retain skilled nurses (22). Identifying professional life quality and associated aspects is one of the most promising approaches to encouraging nurse recruitment and retention, reducing shortage, burnout, and turnover (22). The relationships between areas of job satisfaction among nurses including pay, promotion, supervision, fringe benefits, contingent rewards, operating procedures, coworkers, nature of work, communication, and areas of professional quality of life including burnout, compassion fatigue, and compassion satisfaction have received little attention in prior global literature. Therefore, this research aims to bridge this empirical gap. Moreover, limited studies in Jordan investigated compassion satisfaction and compassion fatigue among nurses, and they were directed to particular groups like intensive care unit nurses or emergency department nurses (23, 24). Thus, this study aimed to examine the association between professional quality of life and job satisfaction among registered nurses working in different units in Jordanian hospitals.

## Methods

### Design

A descriptive correlational cross-sectional design was utilized in this study.

### Sample

A convenient sample design was used to collect study data. An online self-administered questionnaire using Google Forms was utilized. The sample size was estimated using G*Power 3.1.10 (25) based on F statistics, medium effect size =0.25, *α* <0.05, and power of 0.9; the minimum estimated sample size was 300 nurses. Inclusion criteria were: (1) Being a full-time registered nurse; (2) Having at least 1 year of experience.

### Setting

Data were collected from nurses employed in Jordanian governmental, private, and university-affiliated hospitals in Amman, the capital of Jordan. The highest-bed capacity hospitals were selected.

### Ethical considerations

Ethical approval was obtained from the Scientific and Research Committee at the Faculty of Nursing at Al Ahliyya Amman University (No. A2023/1520). Participation was voluntary, and nurses were assured that their responses would be confidential. The anonymity of the participants was ensured throughout the study. The front page of the questionnaire contains the study objectives, confidentiality issues, and the anonymity and privacy of the respondents. Data was secured in a password-protected computer. The study instruments are permitted for public use by the tool developers.

### Data collection

Data collection was begun after obtaining ethical approval. Data was collected using an electronic self-administered questionnaire using Google Forms and was distributed online via e-mails, and social media (WhatsApp, Facebook). The questionnaire contains a description that provides the participant with information about the study purpose, data collection procedure, and rights of the participants, followed by a consent statement that must be checked for acceptance if the participant accepts to participate in the study before answering the instrument questions.

### Measurements

*The demographic information section* contained eight items to collect data on selected demographic variables: age, gender, marital status, work sector, job title, years of experience, department, and shift system.

*The Professional Quality of Life Scale (ProQOL)-Health* by Stamm (5) was used in this study. Professional Quality of Life (ProQOL) is intended for any helper, including health care professionals, social service workers, teachers, attorneys, emergency response, etc. Charles Figley created the measure in the late 1980s under the name Compassion Fatigue Self-Test; then, the ProQOL 5th version was created in 2009. This scale aims to understand the positive and negative aspects of helping those who experience trauma and suffering and can improve the ability to help them and keep their balance. ProQOL is the most used measure of the negative and positive effects of helping others who experience suffering and trauma. The ProQOL has sub-scales for compassion satisfaction (CS), burnout (BO), and compassion fatigue (CF). It consists of 30 items, a five-point Likert scale from 1 (never) to 5 (very often). The tool was found to be valid and reliable, with Cronbach’s alpha values of 0.88 for CS, 0.75 for BO, and 0.81 for CF (5). There are three steps to scoring the ProQOL. The first step is to reverse some items. The second step is to sum the items by subscale and the third step is to convert the raw score to a t-score. Raw scores between 10 and 50 are presented for the three subscales (1) compassion satisfaction, (2) burnout, and (3) compassion fatigue. Each subscale score is also presented as two percentile ranks, comparing the respondent’s scores to typical patterns of responding. And a percentile of 50 represents an average score (5).

*The Job Satisfaction Survey (JSS)* by Paul Spector (26) is a 36-item, nine-subscales to assess employee attitudes about the job. Each subscale is assessed with four items; a total score is computed from all items. A summated rating scale format is used, with a six-point Likert scale ranging from 1 (strongly disagree) to 6 (strongly agree). About half of the items require reverse scoring because they are worded in both ways. The nine subscales are pay, promotion, supervision, fringe benefits, contingent rewards (performance-based rewards), operating procedures (required rules and procedures), coworkers, nature of work, and communication. The internal consistency reliability (coefficient alpha) score for JSS was documented by Spector (27) as 0.91. The instrument has been freely published online for the public to use since 1994.

### Translation of instruments

The current study translated the ProQOL-Health and JSS into Arabic. Brislin’s (54) translation model was applied. The instruments were translated into Arabic from the original language, English, using five steps. The first step involved forward translation involving two native Arabic speakers with Ph.D in nursing. One native Arabic speaker who was not involved in the forward translation reconciled the translations. Then, a native English speaker who was proficient in Arabic and was not engaged in the earlier stages translated the reconciled version back into English from Arabic. After that, the translated and original English versions were compared.

### Validity and reliability

A pilot study was conducted with 15 participants to evaluate the translation instruments’ reliability, the clarity of the language, and how long it took to complete the instruments. The coefficient alpha score for ProQOL was 0.74 and 0.91 for JSS.

### Data analysis

Data were analyzed using the Statistical Package for the Social Sciences version 17 (55). Descriptive statistics were calculated to describe demographic information and the questionnaire items. *T*-test and ANOVA statistics were calculated to detect differences in nurses’ responses concerning their demographics. Multiple logistic regressions were used to identify the predictors of nurses’ professional quality of life.

## Results

### Sample characteristics

A total of 542 nurses participated in the study. The mean age was 32.77 (SD = 7.85), ranging from 22 to 63 years. Nurses’ experience ranged from one to 38 years, with a mean of 9.95 (SD = 7.84). The sample included 74.4% females (*n* = 403), and 60% of the sample (*n* = 325) were married. Most nurses were from private hospitals, 57.4% (*n* = 311). Table 1 shows other characteristics of the sample.

**Table 1:** Sample characteristics (*n* = 542).

Characteristics	*N*	Percentage	Mean (SD)
Age			32.77 (7.85)
Years of experience			9.95 (7.84)
Gender			
Male	139	25.6	
Female	403	74.4	
Social status			
Married	325	60	
Single	191	35.2	
Divorced	19	3.5	
Widowed	7	1.3	
Hospital type			
Public	129	23.8	
Private	311	57.4	
Military	46	8.5	
University-affiliated	56	10.3	
Department			
Intensive and intermediate care units	75	13.8	
Specialized units (endoscopy, catheterization, dialysis, lithotripsy, etc.)	28	5.2	
Medical and Surgical departments	103	19	
Maternity and postpartum departments	65	12	
Emergency department	52	9.6	
Pediatric department	26	4.8	
Operation room	63	11.6	
Others	130	24	
Current position			
Head of department/ Nurse supervisor	4	0.7	
Registered nurse	300	55.4	
Continuous education department	12	2.2	
In charge nurse	57	10.5	
Associate nurse	93	17.2	
Administrative position	76	14	
The shifts you work most often			
Morning shift (A)	247	45.6	
Evening shift (B)	34	6.3	
Night shift (C)	21	3.9	
Rotating shift (A, B, and C)	240	44.3	

### Nurses’ job satisfaction

The mean total score for nurses’ job satisfaction survey was 117.47 (SD = 27.26). The scores ranged between 51 and 212. The Nature of Work subscale had the highest mean, 18.31 (SD = 4.37), while the Fringe Benefits subscale had the lowest mean, 10.28 (SD = 4.05), see Table 2.

**Table 2:** Nurses’ job satisfaction (*N* = 542).

Scale	Mean (SD)	Range
Job satisfaction survey total score	117.47 (27.26)	51–212
Job satisfaction subscales		
Pay subscale	10.59 (4.72)	4–24
Promotion	11.57 (4.33)	4–24
Supervision	15.57 (4.78)	4–24
Fringe benefits	10.28 (4.05)	4–24
Contingent rewards	10.49 (4.69)	4–24
Operating procedures	11.35 (3.05)	4–24
Coworkers	14.56 (4.41)	4–24
Nature of work	18.31 (4.37)	4–24
Communication	14.70 (4.54)	4–24

### Professional quality of life for health workers

The mean total score for the ProQOL-Health was 98.41 (SD = 12.15). The scores ranged between 46 and 142. The compassion satisfaction subscale had the highest mean score of 23.52 (SD = 4.82), while the moral distress subscale had the lowest mean score of 16.44 (4.42), see Table 3.

**Table 3:** Professional quality of life for health workers (*N* = 542).

Scale	Mean (SD)	Range	*N* (%)
Professional quality of life scale for health workers	98.41 (12.15)	46–142	
Compassion satisfaction	23.52 (4.82)	6–30	
Low			18 (3.3)
Average			221 (40.8)
High			303 (55.9)
Perceived support	18.7 (4.57)	7–30	18.7 (4.57)
Low			44 (8.1)
Average			416 (76.8)
High			82 (15.1)
Burnout	20.69 (5.13)	6–30	
Low			34 (6.3)
Average			346 (63.8)
High			162 (29.9)
Secondary traumatic stress	19.05 (4.55)	7–30	
Low			38 (7)
Average			409 (75.5)
High			95 (17.5)
Moral distress	16.44 (4.42)	6–29	
Low			101 (18.6)
Average			411 (75.8)
High			30 (5.5)

### The relationships between nurses’ job satisfaction, professional quality of life for health workers and sociodemographic variables

There was a statistically significant positive relationship between nurses’ job satisfaction and professional quality of life for health workers (*r* = 0.23, *p* < 0.001). There was a significant difference in nurses’ job satisfaction in relation to hospital type (*F* (3, 539) = 13.37, *p* < 0.001). Nurses in private hospitals (*m* = 122.79) reported significantly higher job satisfaction than nurses in public hospitals (*m* = 106.55), *p* < 0.001, and nurses in university-affiliated hospitals (*m* = 110.05), *p* = 0.005. There was a significant difference in nurses’ job satisfaction in relation to work shifts (*F* (3, 539) = 3.57, *p* = 0.014). Nurses working on the morning shift (A) (*m* = 120.30) reported significantly higher job satisfaction than nurses working on the night shift (C) (*m* = 102.86), *p* = 0.024. No significant differences were found between nurses’ job satisfaction and other variables. No significant differences were found between professional quality of life for health workers and sociodemographic variables.

## Discussion

The current study findings provide an essential understanding of exploring the relationship between professional quality of life and job satisfaction among registered nurses working in different units in Jordanian hospitals. The findings give stakeholders a thorough understanding of nurses’ professional quality of life, enlightening appropriate management and motivation techniques to enhance their working conditions, which would thus help to improve their ProQOL, job satisfaction, quality of healthcare, and the efficiency of the healthcare system. The results of this study showed a high total mean score of job satisfaction among nurses, with a mean score of 98.41. This result is similar to other studies done by Kim et al. (28), Niu et al. (29), and Ndlovu et al. (30), and it is higher than was reported by other studies (7, 31, 32). In the present study, the results of ProQOL subdomains were similar to those of the Niu et al. (29) study. The results of compassion satisfaction, burnout, and secondary traumatic stress were better than other studies done in Saudi Arabia, Egypt, and China (32–34). This study’s results may be explained by the improvement in the Jordanian healthcare system, especially after the COVID-19 pandemic when the nurses appeared as the first-line workforce who worked with patients. So, the Jordanian Ministry of Health paid attention to the status of nurses, including improving the quality of their work lives. Another reason for the previous results may be that most of the nurses who participated in this study were from the private sector, where the environment is more comfortable and aided by high technology and resources that ease the work of the nurses (35). Perceived support and moral distress were the lowest-ranked items of ProQOL, possibly due to demanding daily schedules, heavy workloads, strained professional relationships, and emotional difficulties (36). At the same time, perceived support and moral distress were moderate in mean scores, which reflects the growing efforts of hospital administration to support the nurses and deal with organizational obstacles that may contribute to their feelings of moral distress. Counseling sessions that can control moral distress outcomes should be given to nurses. This should be done through a continuous screening process involving routine screening of nurses to detect moral distress (37). The hospital’s administration and the Jordanian Ministry of Health should also put measures in place to address the causes of moral distress. To be relevant, they need to keep investigating the characteristics of moral pain among nurses, coming up with plans that enable nurses to proactively recognize the indicators of moral suffering before it develops into moral distress or burnout, and testing and funding efficient programs for helping those nurses who are going through it (38). In addition, hospitals’ administration could support nurses in being more morally effective which seems to lead to more voice and less moral distress. Also increasing support for organizational ethics might be a crucial strategy (39).

The present study results regarding job satisfaction show a better mean score than many other Jordanian studies (23, 40, 41). This may indicate an improved work life and environment (42). The nature of the work subscale had the highest mean score. However, the lowest mean score was for fringe benefits. This result is supported by studies by Ghazi and Dhafer (43), Loh et al. (56), and Saleh et al. (44). The results show that the healthcare organization’s work environment and benefits should be revised to promote favorable outcomes and improve the overall satisfaction of nurses. This entails implementing a more effective system that uses operating procedures and supports higher satisfaction levels. Hospital managers are advised to implement new reimbursement policies acknowledging nurses’ excellent performance and productivity.

A statistically significant positive correlation between nurses’ job satisfaction and professional quality of life was detected in this study. This result is similar to many other studies (4, 23, 45). This is expected because high professional work-life quality is a good measure of workers’ job satisfaction (46). This study showed a significant difference in nurses’ job satisfaction with hospital type. Nurses in private hospitals reported significantly higher job satisfaction than in public hospitals and university-affiliated hospitals. Most likely, these results may be due to nurses at private hospitals solely carrying out the tasks outlined in their job descriptions. This result is also supported by Geta et al. (47), who explained that private hospitals have better work environments, infrastructure, and resources, which may influence the nature of the work among nurses and, accordingly, will raise job satisfaction.

The current study revealed that Jordanian nurses who participated in this study who are working on the fixed morning shift (A) reported significantly higher job satisfaction than nurses working on the night shift (B) and (C), or rotating shifts, which is similar to many other studies (48, 49). These results may be explained by many reasons, including hospital nurses who work in B, C, and rotating shifts may experience anxiety and stress (50), disruptions in their personal lives (51), disruptions in their regular eating routines, and lower job satisfaction (4, 23).

Measuring job satisfaction among nurses can be challenging because they take their responsibilities seriously and are quite effective at the biomedical parts of nursing care delivery. However, they may be hesitant or even afraid to interact with patients and other healthcare professionals. This group of nurses benefits from encouragement to strengthen their compassion and conviction that they give their patients high-quality care. Also, the moral aspect of their life should be assessed and evaluated occasionally by nurse administrators or specialized healthcare professionals to support them in solving their moral distress and provide them with specific techniques to ease their distress (39). Establishing an efficient management strategy is advised to decrease burnout in a way that decreases stress and raises compassionate satisfaction for nurses. Moreover, the statistically high turnover rate in the nursing profession can be explained by the tremendous strain of the occupation (52). The strain of daily obligations persists even after the COVID-19 pandemic. In this study results one of the lower satisfaction was related to pay scale compared to other factors. So, offering decent salaries is a great place to start (53). It is one of the main motivations for many people’s first decision to become nurses and a motivator for improving satisfaction. For the most part, though, nursing is about more than money. Their concerns are career growth, general well-being, and professional work-life balance. Offering fringe benefits that are truly helpful to nurses is a great way to express the organization’s gratitude and lighten their overload. This study recommended that nursing supervisors be more aware of the variables influencing nurses’ professional quality of life and job to assess the degree of moral distress and satisfaction.

Although nurses offer their patients physical, psychological, and spiritual care, their duties and interactions with patients can negatively affect them. As a result, nurses will be better equipped to care for patients if they have the assistance and support they need. Several limitations of this study need to be acknowledged. Job satisfaction was correlated with work-related stress, burnout, compassionate satisfaction, support, and nurses’ moral distress; however, many other variables could interfere with the results, such as personal stressors like family issues, financial situations, and dysfunctional relationships. So, in future studies, it is recommended that many other variables be addressed using different tools. Another limitation is convenient sampling, so the random sampling strategy can be used in future studies to guarantee that everyone has an equal opportunity to be included in the research and that the recruited sample represents the population under investigation. These limitations call for more longitudinal studies to examine the mediation effects of job satisfaction and ProQOL among nurses.

This study has various merits, including that it is the first study done in Jordan that combines job satisfaction with the ProQOL. In addition, the findings better reflect the involvement of nurses from various units. More studies are advised to use this strategy in various clinical settings and conduct a long-term analysis of ProQOL over a long period.

## Conclusion

This study shows a significantly positive correlation between job satisfaction and nurses’ professional quality of life in Jordan. This study also showed that nurses who work in the private sector and in permanent A shift experience job satisfaction more than others. Promoting more stress-free, motivated, enough payment and incentives working conditions that lower health system costs and inefficiencies while raising patient outcomes, employee happiness, and quality of treatment is necessary to increase the nurses’ professional quality of life and satisfaction.

Creating a morally stress-free and healthy work environment is something that legislators and nurse managers should prioritize to deliver higher-quality services. First and foremost, research into the variables influencing nursing professionals’ professional quality of life and the healthcare services that have the most significant impact on or disrupt their well-being should be the focus of future studies. Therefore, employment and organizational measures have to be put into place appropriately. Targeted interventions should be developed depending on the situations that put nurses in moral distress. The goal of these interventions ought to be to foster adaptation and stress release. Counseling and supporting nursing staff are necessary programs that should be considered in Jordan hospitals.

## Data Availability

The raw data supporting the conclusions of this article will be made available by the authors, without undue reservation.

## References

[1] World Health Organization (2022). Nursing and midwifery. Available at: https://www.who.int/news-room/fact-sheets/detail/nursing-and-midwifery (Accessed May 15, 2024).

[2] Akter N Akter MK Turale SOUTH. Barriers to quality of work life among Bangladeshi nurses: a qualitative study. Int Nurs Rev. (2019) 66:396–403. doi: 10.1111/inr.12540, PMID: 31393005

[3] Ozkara San E. Concept analysis of nurses' happiness. Nurs Forum. (2015) 50:55–62. doi: 10.1111/nuf.12099, PMID: 24935713

[4] Javanmardnejad S Bandari R Heravi-Karimooi M Rejeh N Sharif Nia H Montazeri A. Happiness, quality of working life, and job satisfaction among nurses working in emergency departments in Iran. Health Qual Life Outcomes. (2021) 19:1–8. doi: 10.1186/s12955-021-01755-3 33794917 PMC8017644

[5] Stamm BH. The concise ProQOL manual. Pocatello, ID: ProQOL (2010).

[6] Kakemam E Gharaee H Rajabi MR Nadernejad M Khakdel Z Raeissi P. Nurses’ perception of patient safety culture and its relationship with adverse events: a national questionnaire survey in Iran. BMC Nurs. (2021) 20:60. doi: 10.1186/s12912-021-00571-w, PMID: 33845822 PMC8042945

[7] Ruiz-Fernández MD Pérez-García E Ortega-Galán ÁM. Quality of life in nursing professionals: burnout, fatigue, and compassion satisfaction. Int J Environ Res Public Health. (2020) 17:1253. doi: 10.3390/ijerph17041253, PMID: 32075252 PMC7068555

[8] Mottaghi S Poursheikhali H Shameli L. Empathy, compassion fatigue, guilt and secondary traumatic stress in nurses. Nurs Ethics. (2020) 27:494–504. doi: 10.1177/0969733019851548, PMID: 31284826

[9] Scott SD Hirschinger LE Cox KR McCoig M Brandt J Hall LW. The natural history of recovery for the healthcare provider “second victim” after adverse patient events. Qual Saf Health Care. (2009) 18:325–30. doi: 10.1136/qshc.2009.032870, PMID: 19812092

[10] Yildirim JG Ertem M. Professional quality of life and perceptions of spirituality and spiritual care among nurses: relationship and affecting factors. Perspect Psychiatr Care. (2022) 58:438–47. doi: 10.1111/ppc.12794, PMID: 33834515

[11] Grelier A Guerin O Levavasseur F Caillot F Benichou J Caron F. Personal and professional quality of life among French health care workers during the first COVID-19 wave: a cross-sectional study. BMC Nurs. (2022) 21:80. doi: 10.1186/s12912-022-00860-y, PMID: 35392883 PMC8986964

[12] Sundgren MKM Millear PM Dawber C Medoro L. Reflective practice groups and nurse professional quality of life. Aust J Adv Nurs. (2021) 38:355. doi: 10.37464/2020.384.355

[13] Mills J Wand T Fraser JA. Examining self-care, self-compassion and compassion for others: a cross-sectional survey of palliative care nurses and doctors. Int J Palliat Nurs. (2018) 24:4–11. doi: 10.12968/ijpn.2018.24.1.4, PMID: 29368553

[14] Raab K. Mindfulness, self-compassion, and empathy among health care professionals: a review of the literature. J Health Care Chaplain. (2014) 20:95–108. doi: 10.1080/08854726.2014.913876, PMID: 24926896

[15] Hesam M Asayesh H Roohi G Shariati A Nasiry H. Assessing the relationship between nurses’ quality of work life and their intention to leave the nursing profession. Q J Nurs Manag. (2012) 1:28–36.

[16] Zarei E Ahmadi F Danshkohan A Ramezankhani A. The correlation between organizational commitment and the quality of working life among staff of Sarpolzahab health network. J Health Promot Manag. (2016) 5:61–9.

[17] Davey BR Byrne SJ Millear PM Dawber C Medoro L. Evaluating the impact of reflective practice groups for nurses in an acute hospital setting. Aust J Adv Nurs. (2021) 38:220. doi: 10.37464/2020.381.220

[18] Lee E Daugherty JA Eskierka K Hamelin K. Compassion fatigue and burnout, one Institution’s interventions. J Perianesth Nurs. (2019) 34:767–73. doi: 10.1016/j.jopan.2018.11.003 30773407

[19] Sarafis P Rousaki E Tsounis A Malliarou M Lahana L Bamidis P. The impact of occupational stress on nurses’ caring behaviors and their health related quality of life. BMC Nurs. (2016) 15:56. doi: 10.1186/s12912-016-0178-y, PMID: 27708546 PMC5039891

[20] Simon CE Pryce JG Roff LL Klemmack D. Secondary traumatic stress and oncology social work: protecting compassion from fatigue and compromising the worker’s worldview. J Psychosoc Oncol. (2005) 23:1–14. doi: 10.1300/J077v23n04_01 16618685

[21] Sung K Seo Y Kim JH. Relationships between compassion fatigue, burnout, and turnover intention in Korean hospital nurses. J Korean Acad Nurs. (2012) 42:1087–94. doi: 10.4040/jkan.2012.42.7.1087, PMID: 23377605

[22] Phan GT Vo TQ. A literature review on quality of working life: a case of healthcare workers. J Appl Pharm Sci. (2016) 6:193–200. doi: 10.7324/JAPS.2016.60729

[23] Salahat MF Al-Hamdan ZM. Quality of nursing work life, job satisfaction, and intent to leave among Jordanian nurses: a descriptive study. Heliyon. (2022) 8:e09838. doi: 10.1016/j.heliyon.2022.e09838, PMID: 35815152 PMC9260616

[24] Suleiman K Hijazi Z Al Kalaldeh M Abu Sharour L. Quality of nursing work life and related factors among emergency nurses in Jordan. J Occup Health. (2019) 61:398–406. doi: 10.1002/1348-9585.12068, PMID: 31215754 PMC6718837

[25] Faul F Erdfelder E Lang A-G Buchner A. G*power 3: a flexible statistical power analysis program for the social, behavioral, and biomedical sciences. Behav Res Methods. (2007) 39:175–91. doi: 10.3758/BF03193146 17695343

[26] Spector PE. Measurement of human service staff satisfaction: development of the job satisfaction survey. Am J Community Psychol. (1985) 13:693–713. doi: 10.1007/BF00929796 4083275

[27] Spector PE. Job satisfaction: Application, assessment, causes, and consequences. Thousand Oaks, CA: Sage (1997).

[28] Kim K Han Y Kwak Y Kim JS. Professional quality of life and clinical competencies among Korean nurses. Asian Nurs Res. (2015) 9:200–6. doi: 10.1016/j.anr.2015.03.002, PMID: 26412623

[29] Niu A Li P Duan P Ding L Xu S Yang Y. Professional quality of life in nurses on the frontline against COVID-19. J Nurs Manag. (2022) 30:1115–24. doi: 10.1111/jonm.13620, PMID: 35403339 PMC9115241

[30] Ndlovu E Filmalter C Jordaan J Heyns T. Professional quality of life of nurses in critical care units: influence of demographic characteristics. South Afr J Crit Care. (2022) 38:39–43. doi: 10.7196/SAJCC.2022.v38i1.517, PMID: 35814622 PMC9258033

[31] Jang I Kim Y Kim K. Professionalism and professional quality of life for oncology nurses. J Clin Nurs. (2016) 25:2835–45. doi: 10.1111/jocn.13330 27335236

[32] Mokadem NE Shimaa ES. The relationship between work environment and nurses’ professional quality of life in critical care settings. Int J Novel Res Healthc Nurs. (2020) 7:76–83.

[33] Cruz JP Alquwez N Mesde JH AidAlmoghairi AM Altukhays AI Colet PC. Spiritual climate in hospitals influences nurses' professional quality of life. J Nurs Manag. (2020) 28:1589–97. doi: 10.1111/jonm.13113, PMID: 32743944

[34] Tian MM Fan L Shi Y Wei XM Jiang H Wu Y. The current status and influencing factors of compassion fatigue in clinical nurses. Chin J Nurs. (2018) 53:76–82. doi: 10.3761/j.issn.0254-1769.2018.01.014

[35] Ullah H Sehrish A Anwar CH Rana SM. Factors influencing job satisfaction of nurses in public and private Sector's hospitals: a cross sectional study. Pak J Public Health. (2018) 8:147–51. doi: 10.32413/pjph.v8i3.177

[36] Austin CL Saylor R Finley PJ. Moral distress in physicians and nurses: impact on professional quality of life and turnover. Psychol Trauma. (2017) 9:399–406. doi: 10.1037/tra0000201, PMID: 27797570

[37] Villani D Grassi A Cognetta C Toniolo D Cipresso P Riva G. Selfhelp stress management training through mobile phones: an experience with oncology nurses. Psychol Serv. (2013) 10:315–22. doi: 10.1037/a0026459, PMID: 23937091

[38] Rushton CH. Moral resilience: a capacity for navigating moral distress in critical care. AACN Adv Crit Care. (2016) 27:111–9. doi: 10.4037/aacnacc2016275, PMID: 26909461

[39] Rathert C May DR Chung HS. Nurse moral distress: a survey identifying predictors and potential interventions. Int J Nurs Stud. (2016) 53:39–49. doi: 10.1016/j.ijnurstu.2015.10.007, PMID: 26515025

[40] Hamaideh SH. Burnout, social support, and job satisfaction among Jordanian mental health nurses. Issues Ment Health Nurs. (2011) 32:234–42. doi: 10.3109/01612840.2010.546494, PMID: 21355758

[41] Al-Hamdan Z Manojlovich M Tanima B. Jordanian nursing work environments, intent to stay, and job satisfaction. J Nurs Scholarsh. (2017) 49:103–10. doi: 10.1111/jnu.12265, PMID: 27899008

[42] Alabed Hasan RWEM. Work environment and job satisfaction among nurses in Jordan: a systematic literature review. Br J Healthc Manag. (2023) 29:1–7. doi: 10.12968/bjhc.2021.0128

[43] Ghazi Baker O Dhafer Alshehri B. The relationship between job stress and job satisfaction among Saudi nurses: a cross-sectional study. Nurse Media J Nurs. (2020) 10:292–305. doi: 10.14710/nmjn.v10i3.32767

[44] Saleh AM Saleh MM AbuRuz ME. The impact of stress on job satisfaction for nurses in king Fahad specialist hospital-Dammam-KSA. J Am Sci. (2013) 9:371–7.

[45] Morsy SM Sabra HE. Relation between quality of work life and nurses job satisfaction at Assiut university hospitals. Al-Azhar Ass Med J. (2015) 13:163–71.

[46] Hsu M-Y. A quality of working life survey instrument for hospital nurses. J Nurs Res. (2016) 24:87–99. doi: 10.1097/jnr.0000000000000098 26309118

[47] Geta A Biks GA Dellie E Yazachew L. Job satisfaction and associated factors among health professionals working at public and private hospitals in Bahir Dar City, Northwest Ethiopia: a comparative cross-sectional study. Biomed Res Int. (2021) 2021:1–13. doi: 10.1155/2021/6632585, PMID: 34493980 PMC8418930

[48] Acea-López L Pastor-Bravo MDM Rubinat-Arnaldo E Bellon F Blanco-Blanco J Gea-Sanchez M. Burnout and job satisfaction among nurses in three Spanish regions. J Nurs Manag. (2021) 29:2208–15. doi: 10.1111/jonm.13376, PMID: 33998728

[49] Lu H Zhao Y While A. Job satisfaction among hospital nurses: a literature review. Int J Nurs Stud. (2019) 94:21–31. doi: 10.1016/j.ijnurstu.2019.01.011 30928718

[50] Moradi S Farahnaki Z Akbarzadeh A Gharagozlou F Pournajaf A Abbasi AM. Relationship between shift work and job satisfaction among nurses: a cross-sectional study. Int J Hosp Res. (2014) 3:63–8.

[51] Jaradat YM Nielsen MB Kristensen P Bast-Pettersen R. Shift work, mental distress and job satisfaction among Palestinian nurses. Occup Med. (2017) 67:71–4. doi: 10.1093/occmed/kqw128, PMID: 27694376 PMC5225884

[52] Zhang YY Han WL Qin W Yin HX Zhang CF Kong C. Extent of compassion satisfaction, compassion fatigue and burnout in nursing: a meta-analysis. J Nurs Manag. (2018) 26:810–9. doi: 10.1111/jonm.12589, PMID: 30129106

[53] Baljoon RA Banjar HE Banakhar MA. Nurses’ work motivation and the factors affecting it: a scoping review. Int J Nurs Clin Pract. (2018) 5:277. doi: 10.15344/2394-4978/2018/277

[54] Brislin RW. (1986). The wording and translation of research instrument. In Lonner W. J. Berry J. W. (Eds.), Field methods in cross-cultural research (pp. 137–164). Beverly Hills, CA: Sage.

[55] IBM Corp. Released. (2012). IBM SPSS Statistics for Windows, Version 21.0. Armonk, NY: IBM Corp.

[56] Loh HS Gan LY Lim ZW Loh WS Yong SY. (2016). The relationship between Stress and Job Satisfaction of Nurses in private hospitals of Georgetown Penang (Doctoral dissertation UTAR).

